# Thermal-Assisted Laser Fabrication of Broadband Ultralow Reflectance Surface by Combining Marangoni Flow with In Situ Deposition

**DOI:** 10.3390/nano13030480

**Published:** 2023-01-25

**Authors:** Jingbo Yin, Huangping Yan, Rui Zhou, Yuanzhe Li, Anna He

**Affiliations:** 1 School of Aerospace Engineering, Xiamen University, Xiamen 361005, China; 2 Shenzhen Research Institute of Xiamen University, Shenzhen 518000, China

**Keywords:** anti-reflection, Marangoni effect, in situ deposition, thermal-assisted laser fabrication, multi-scale structure

## Abstract

Functional surfaces with broadband ultralow optical reflectance have many potential applications in the fields of enhancing solar energy utilization, stray light shielding, infrared stealth, and so on. To fabricate broadband anti-reflection surfaces with low cost, high quality, and more controllability, a strategy of preparing multi-scale structures by thermal-assisted nanosecond laser was proposed. This strategy combines laser ablation with Marangoni flow of molten materials and in situ deposition of nanoparticles. The thermal-assisted strategy increases the depth to width ratio of the anti-reflection structures. The average reflectance of laser-textured TC4 (Ti-6Al-4V) surface is as low as 1.71% in the wavelength range of 200–2250 nm and 7.8% in the 2500–25,000 nm. The ultra-low reflectance surface has a significantly enhanced photothermal conversion performance. Meanwhile, the anti-reflection effect can be extended to the mid-infrared band, which has potential stealth application prospect. This synergetic manufacturing strategy has wide adaptability of materials, which provides new paths for the preparation of broadband ultralow reflectance surface. Moreover, this thermal-assisted laser fabrication strategy is prospective in the preparation of other functional micro-nano structures.

## 1. Introduction

In the face of an energy crisis and resource shortages, solar energy, as a permanent and universal energy source, has become one of the keys to solve the problem. Solar energy is widely used in photothermal conversion [1,2,3], photovoltaic devices [4,5,6], and so on. Ultra-strong light absorption interface can greatly improve the energy utilization rate. Reducing the reflection of the material surface in a wider wavelength band can be applied to artificial blackbody [7], stray light shielding [8], infrared stealth [9,10], and infrared imaging [11]. Titanium alloy is widely used in medical life, national defense, aerospace, and other fields because of its low density, high specific strength, and high temperature resistance [12,13]. Due to the high optical impedance, common coating strategies usually cannot effectively bridge the refractive gap between the metal surface and the air [14]. Various surface morphology give the surface unique optical properties [15], wetting properties [16,17], etc. Therefore, obtaining broadband light absorption surface through surface micro-nano structure has become a hot research topic.

Pulsed laser has been proven to be a powerful tool for fabricating micro-nano structures on different materials [18,19,20]. By adjusting the femtosecond laser flux, scanning interval, and polarization, Li et al. prepared disordered titanium alloy surface with ultra-low average reflectance of 2% in the spectral band of 250–2300 nm [21]. Lian et al. prepared disordered anti-reflection microstructure on the TC4 surface by nanosecond laser. The average reflectance of the sample in the wavelength range of 500–1000 nm is stable below 6%, and the static contact angle of the sample reaches 150° [22]. Some researchers adopted two-step laser processing to achieve low reflectance surface [23,24,25]. Cheng et al. combined nanosecond and femtosecond pulse lasers to prepare TC4 surface with an average reflectance of 3.1% in the wavelength range of 250–2300 nm [25].

Laser processing not only gives metal surface unique optical properties but also has potential applications in changing the wettability of materials [26,27,28]. However, when the microcolumn structure is prepared by laser ablation milling, the size of light spot determines the minimum distance between adjacent microcolumns. This reduces the distribution density of the microcolumns, decreasing the duty ratio and limiting its anti-reflection performance. Interestingly, the size of recast layer formed by phase explosion and Marangoni flow during laser processing is much smaller than the spot size [29,30]. Meanwhile, the distribution of recast layer formed by solidification of molten material is related to scanning path. By adjusting the laser parameters, the molten material can be distributed regularly.

In this paper, a strategy by thermal-assisted nanosecond laser processing is proposed to fabricate anti-reflection surface. Multi-scale micro-nano structures with high duty ratio were formed by Marangoni flow and in situ deposition to redistribute the molten material on TC4 surface regularly. The multi-scale structure prepared by one-step thermal-assisted nanosecond laser processing shows excellent anti-reflection performance in the ultra-wide spectrum. The potential of the thermal-assisted laser fabrication strategy in the preparation of functional micro-nano composite structures was demonstrated. Compared with femtosecond laser and picosecond laser, nanosecond laser has the advantages of low cost and simple maintenance. This simple, low-cost, high-efficiency preparation strategy of broadband anti-reflection structure has a promising application in the field of metal antireflection.

## 2. Experimental

### 2.1. Materials and Methods

The material selected in this experiment was TC4 (Ti-6Al-4V, Dongguan Guangfeng metal material Co. Ltd., Dongguan, China) with the size of 30 mm × 30 mm × 1 mm.

The samples were washed with absolute ethanol and deionized water in ultrasonic cleaning machine (JP-020, Skymen, Shenzhen, China) for 5 min before laser processing, respectively. The power of the ultrasonic cleaning machine is 120 W, and the ultrasonic frequency is 40 KHz. The anti-reflection structure was fabricated by a solid-state laser (Seal-355, JPT, Shenzhen, China) at a pulse duration of 10 ns emitting in the wavelength of 355 nm. The repetition rate of the laser is 40 KHz and the laser fluence is 41.6 J/cm^2^.

### 2.2. Characterization and Measurement

A field emission scanning electron microscope (JSM-IT500A, JEOL, Tokyo, Japan) was used to characterize the surface morphology of multi-scale structure samples. The reflectance spectra of the samples at 200–2250 nm were measured by a spectrophotometer (Lambda 1050+, PerkinElmer, Norwalk, CT, USA) with an integrating sphere accessory. The reflectance spectra of the samples at 2500–25,000 nm were measured by a FTIR spectrometer (Nicolet iS50, Thermo Fisher Scientific, Waltham, MA, USA) with an integrating sphere accessory.

## 3. Results and Discussion

### 3.1. Marangoni Flow and In Situ Deposition

**Figure 1:**
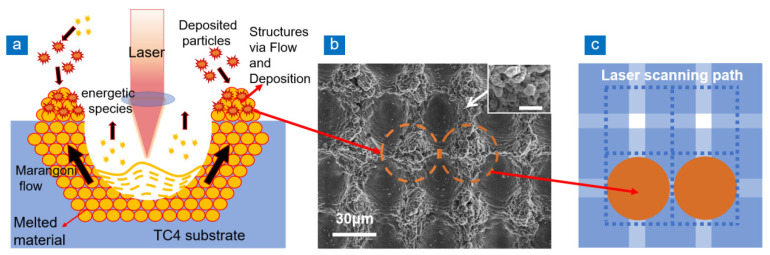
Generation mechanism and SEM diagram of recast layer. (**a**) Generation mechanism of recast layer, (**b**) SEM images of high duty ratio structure of TC4 sample surface (the scale bar in the top-right corner is 1 μm), (**c**) laser scanning path.

During laser processing, many photons are absorbed by electrons of TC4 substrate [31,32]. Following that, the electrons transfer to the crystal lattice in a short time, causing thermal diffusion and increasing the temperature of the target material. This leads to the heating, melting, and evaporation of the material and changes it from solid to liquid or gas, and produces plasma. There is a significant temperature difference between the irradiated area and the nearby unirradiated area. Usually, the surface tension of liquid decreases with the increase of temperature, so shear stress is generated in the cold–hot interface area of the material. The thermal effect of nanosecond laser processing is obvious. Figure 1a shows the formation mechanism of micro-grooves and micro-nano structures on both sides in the laser irradiation area. The thermal effect accumulated on the material surface keeps the ablated area in the molten state and enhances the shear stress at the cold–hot interface, resulting in the Marangoni effect. Marangoni flow drives the material to both sides of the laser irradiation position, eventually forming a ridge structure. Meanwhile, the vapor and plasma plume in the laser-irradiated area cause phase explosion in the crater, which leads to abundant high-energy substances (atoms, clusters, and particles) being discharged laterally. Due to gravity and atmospheric pressure, the cooled energetic species agglomerate into nanoparticles and deposit on the material surface [33,34]. Under the thermal effect, these deposited particles combine with the ridge structure formed by Marangoni flow to form the recast layer composed of micro-nano structure. Figure 1b shows the SEM image of TC4 after laser processing. The recast layer is in the dotted orange ring in the figure, and the diameter of the orange ring is 38 μm. The distance between adjacent rings is very close, far smaller than the laser spot diameter (30 μm). The duty ratio of microcolumn structure prepared by recast layer approaches 1, which is beneficial to anti-reflection. The illustration in the top-right corner of Figure 1b shows the nanoparticles around the recast layer. Figure 1c shows the laser scanning path and the method of obtaining the recast layer with high duty ratio. The blue dotted line is the laser scanning path, and the light blue stripe is the area covered by the laser spot and its heat affected zone. The dark blue area is the overlapping area of light blue stripes. When the distance between adjacent paths is smaller than the width of light blue stripes, the laser overlapping process makes the recast layer tend to be disordered. When the distance between adjacent paths is slightly larger than the width of the light blue stripe, there is a small area (white area) that is not ablated by laser. The white area is less affected by heat and the temperature is lower. Therefore, the Marangoni flow caused by the temperature difference is strong, and the molten material gathers in the non-ablated area to form the recast layer. There are many disordered particles and cluster structures on the recast layer surface, but their distribution positions show high regularity. At first, the molten material mainly accumulated in the white area, and finally became an almost tangential orange recast layer in Figure 1c.

**Figure 2:**
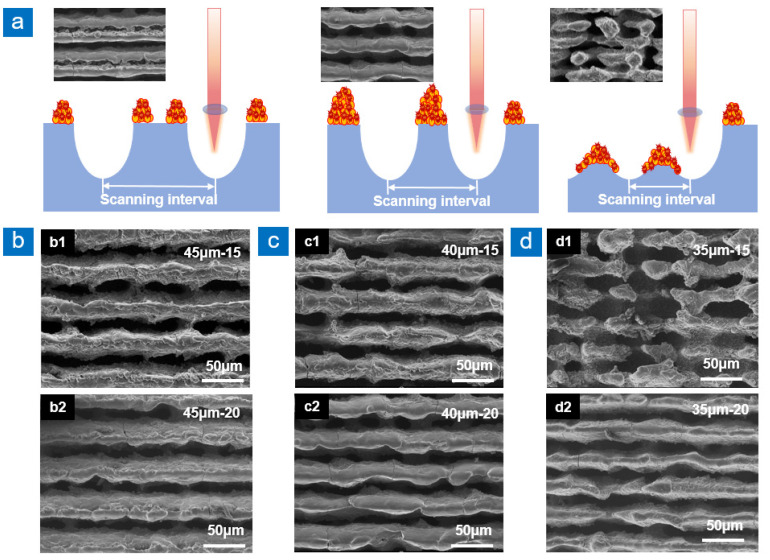
Influence of different scanning intervals on the morphology of recast layer at two sides of groove. (**a**) Schematic diagram of the influence of scanning intervals on recast layer, (**b**) SEM images of TC4 with scanning times of 15 (**b1**) and 20 (**b2**) when the scanning interval is 45 μm, (**c**) SEM images of TC4 with scanning times of 15 (**c1**) and 20 (**c2**) when the scanning interval is 40 μm, (**d**) SEM images of TC4 with scanning times of 15 (**d1**) and 20 (**d2**) when the scanning interval is 35 μm.

Figure 2 shows the influence of scanning interval and scanning times on the distribution of recast layer through parallel scanning. In Figure 2a, when the scanning interval is large, the recast layers on both sides of adjacent scanning paths are far apart, and there is an obvious gap between two independent recast layers. When the scanning interval is appropriate, the recast layers of adjacent scanning paths stack in the non-ablated area to form a larger recast layer, thus increasing the aspect ratio of the groove. When the aspect ratio of the groove is large, the incident light is reflected many times in the groove, and the reflectance is reduced. However, when the scanning interval is too small, there is not enough non-ablated area as supporting area to withstand the molten material, which leads to the disorder of the structure.

As shown in Figure 2b, when the scanning interval is 45 μm, the molten material is regularly distributed in the non-ablated area. Figure 2(b1) shows that when the scanning times are 15, the upward Marangoni flow continues to occur during the groove manufacturing process. The high-energy substances produced by the laser irradiation area are continuously discharged from the grooves, resulting in more disordered particle structures on the recast layer surface. With the increase in scanning times, the groove depth increases. High-energy substances generated at the bottom of the groove are not discharged from the groove, and then gather on the side wall of the groove to form rich micro-nano structures. Figure 2(b2) shows that when the scanning times are 20, the molten material flows under the action of Marangoni convection and gravity flow, which finally makes the top of the recast layer smoother. Figure 2c shows the groove morphology when the scanning interval is 40 μm, which is similar to Figure 2b. When the scanning interval is reduced to 35 μm, the non-ablated area as the supporting area of the molten material is also decreased. When the scanning times are 15, the excess molten material tends to be disordered due to the lack of supporting area, and the recast layer is interrupted, as shown in Figure 2(d1). When the scanning times increase to 20, the heat generated in the laser scanning process makes the recast layer melt and solidify repeatedly, resulting in the continuous smoothness of the recast layer top, as shown in Figure 2(d2).

### 3.2. Preparation of Multi-Scale Microcolumn Structure

When the laser scanning path is parallel-cross line, both the ablation area and the amount of material melting increase. At this time, the non-ablated area is a small columnar structure, and the upper part of the column can not completely withstand the molten material. Excessive molten material flows from the top to the side of the columnar structure under the action of gravity, which significantly increases the radius of the columnar structure. Under the condition of constant period, the radius of the columnar structure increases, which greatly improves the duty ratio. By adjusting the parameters, the distance between microcolumns can be much smaller than the spot size. The small scan interval shortens the structure cycle and increases the number of microcolumns, which leads to an increase in light-trapping area and a decrease in reflectance. It simultaneously reduces the non-ablation area and increases the material melting. When the non-ablated area cannot withstand the molten material, it leads to the disorder of the recast layer, as shown in Figure 2d. Therefore, the scanning intervals of 40 μm and 45 μm are beneficial to the preparation of high duty ratio anti-reflection structures.

**Figure 3:**
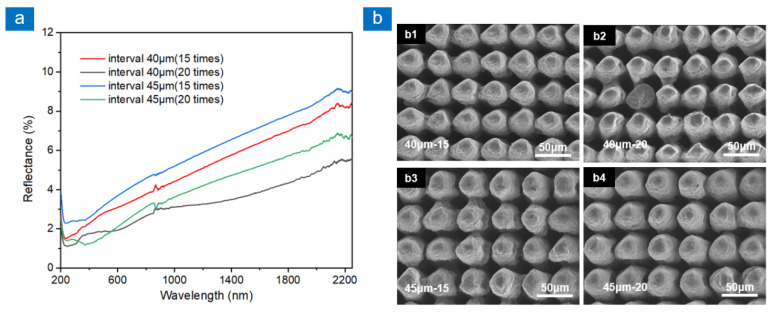
Reflection spectra and SEM images of multi-scale structures at different laser scanning intervals and times. (**a**) Reflectance spectra of four samples, (**b**) SEM images of four samples: (**b1**) the scanning interval is 40 μm and scanning times are 15, (**b2**) the scanning interval is 40 μm and scanning times are 20, (**b3**) the scanning interval is 45 μm and scanning times are 15, (**b4**) the scanning interval is 45 μm and scanning times are 20.

A multi-scale structure composed of microcolumns and surface recast layer was prepared by cross-scanning, as shown in Figure 3. Under two scanning intervals, the microcolumn structures are ordered and nearly tangent, as shown in Figure 3b. The distance between adjacent microcolumns is much smaller than the spot size.

The period of the microcolumn structure decreases when the scan interval is 40 μm. The number of microstructures in the same area increases, so the light-trapping area increases. When the scanning times increase, the depth of microstructure increases. This improves the aspect ratio of the microstructure and enhances its anti-reflection performance. Therefore, as shown in Figure 3a, when the laser scanning interval is 40 μm and the scanning times are 20, the average reflectance of the sample at 200–2250 nm is 3.32%. However, too many scanning times make the microcolumn unable to withstand too much molten material and collapse, thus weakening the light-trapping capacity.

**Figure 4:**
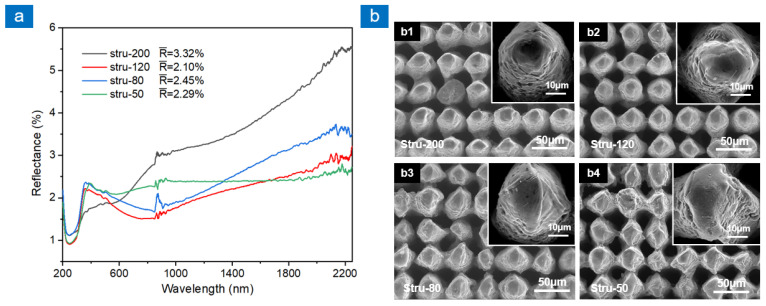
Reflection spectra and SEM images of multi-scale structures at different laser scanning speeds. (**a**) Reflectance spectra of four samples, (**b**) SEM images of four samples: (**b1**) the scanning speeds is 200 mm/s, (**b2**) the scanning speeds is 120 mm/s, (**b3**) the scanning speeds is 80 mm/s, (**b4**) the scanning speeds is 50 mm/s.

All samples in Figure 3 were prepared at a scanning speed of 200 mm/s. When the scanning speed decreases, more heat is accumulated in a single scanning process, which significantly affects the recast layer morphology. The scanning time is varied to 20, 12, 8, and 5 when the laser scanning speed is 200, 120, 80, and 50 mm/s, respectively. Figure 4 shows the reflection spectra and SEM images of multi-scale structures at different scanning speeds. Different scanning speeds have a great influence on the recast layer (Figure 4b), resulting in different reflectance. When the scanning speed is 200 mm/s, the molten material produced by a single scanning process is reduced. Meanwhile, more scanning times make the material melt and solidify repeatedly. This is beneficial to the regularity of the distribution position on the molten materials, therefore the composite structure composed of microcolumn and recast layer is the most regular at this laser parameter, as shown in Figure 4(b1). When the scanning speed is 120 mm/s, the heat accumulation in a single scanning process increases. High-energy substances produced by laser ablation increase and gather on the side of the microcolumn to form a micro-nano composite structure, as shown in Figure 4(b2). This makes stru-120 achieve the lowest reflectance among the four samples, and the average reflectance at 200–2250 nm is 2.10%. As the scanning speed decreases, the molten material produced by single laser scanning increases, which eventually leads to the disordered and rough recast layer, as shown in Figure 4(b3,b4). When the scanning speed was 50 mm/s, too much molten material accumulated between adjacent microcolumns, which reduced the light-trapping area. The reflectance of this sample around 1000 nm wavelength is higher than that of stru-80 and stru-120. There are more disordered nanostructures on the microcolumn surface. When the incident light wavelength is around 1500 nm, the size of most disordered nanostructures is smaller than the incident light wavelength. Based on the effective medium theory, the disordered sub-wavelength structures can form a transition layer with a gradual change in the equivalent refractive index on the microcolumn surface. The transition layer weakens the sudden change of refractive index between the two media and reduces the reflectance of stru-50 in the 1500–2250 nm band. Therefore, unlike the other three samples, the reflectance of stru-50 does not increase significantly around 1500 nm. In turn, the reflectance profile of stru-50 is the most uniform.

### 3.3. Thermal-Assisted Strategy

**Figure 5:**
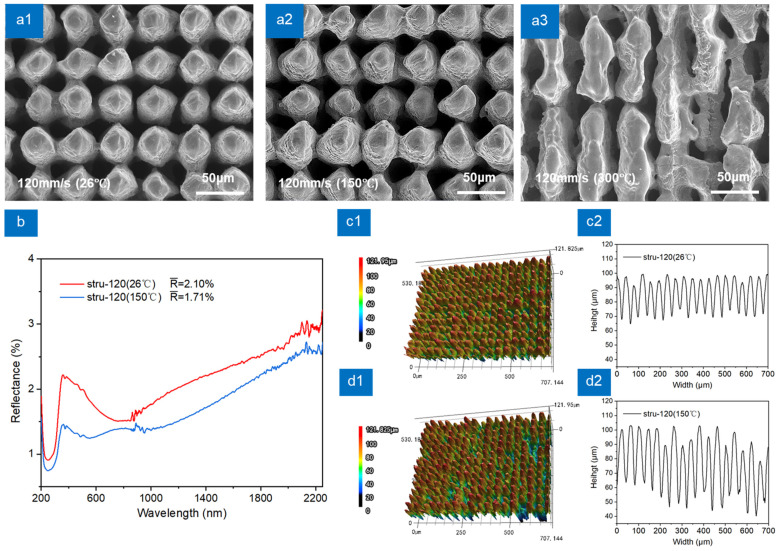
Reflection spectra and topographic images of multi-scale structures at thermal-assisted strategy. (**a1**–**a3**) SEM images of multi-scale structures at different thermal-assisted temperatures: (**a1**) 26 °C, (**a2**) 150 °C, (**a3**) 300 °C, (**b**) Reflectance spectra of stru-120 (26 °C) and stru-120 (150 °C), (**c1**,**c2**) LCM image and cross-sectional view of stru-120 (26 °C), (**d1**,**d2**) LCM image and cross-sectional view of stru-120 (150 °C).

The effect of microstructure morphology on anti-reflection performance is complex and not fully conclusive, but high aspect ratio is a well-recognized factor that can improve the performance of microstructure anti-reflection [35]. All the samples in Figure 3 and Figure 4 were prepared by laser scanning at room temperature (26 °C). A thermal-assisted strategy was proposed to improve the ablation efficiency of the material in order to obtain microstructures with higher aspect ratios at the same laser scanning parameters. TC4 was heated by a heating plate, and laser scanning was carried out during the heating process. The heating temperature of the heating plate is adjustable. The temperature of TC4 sample was measured by infrared thermography. By controlling the temperature of the heating plate, the temperature of TC4 sample was finally stabilized at 0 to 300 °C. When the scanning speed is 120 mm/s and the assisted heating temperature is from 0 to 300 °C, the recast layer tends to be disordered, as shown in Figure 5(a1–a3). When the assisted heating temperature is 300 °C, more molten materials accumulate between the microcolumns, which weakens the light-trapping capacity of the structure. Figure 5b shows that when the assisted heating temperature is 150 °C, the reflectance of stru-120 (150 °C) decreases further than that of stru-120 (26 °C). As shown in Figure 5(c1,d1), thermal-assisted strategy reduces the uniformity of stru-120 (150 °C) and makes the height of microcolumn uneven. The cross-sectional views of the samples shown in Figure 5(c2,d2) were obtained by averaging ten different cross-sections. The distance between adjacent cross-sections is 40 μm. The cross-sectional views represent the overall morphology of the microcolumn arrays. Although the thermal-assisted strategy reduced the uniformity of the microcolumn height, the overall height of the microcolumn arrays remained in a definite range. Meanwhile, the microcolumns of stru-120 (150 °C) have a higher aspect ratio than those of stru-120. Therefore, the reflectance of stru-120 (150 °C) is lower. Compared with the melting point of TC4 (about 1660 °C), the lower heating temperature (below 300 °C) still has a great influence on the morphology of the recast layer. This indicates that the heat from external sources can increase the heat content of the processed sample. It reduces the latent heat of melting and evaporation and makes the materials easier to melt [36].

Thermal-assisted strategy can not only change the microstructure, but also increase the degree of metal oxidation. The existing literature shows that the existence of oxide layer is beneficial to metal anti-reflection [14,24,37]. The elemental composition of the sample surface was characterized by EDS. The atomic number percentages of the two elements were obtained by the mass ratio of Ti to O. The results are shown in Table 1. Due to more scanning times, the Ti:O ratio has reached 38.23:61.77 without thermal assistance. However, the oxidation of Ti is further enhanced by heat-assisted processing, and Ti has been almost completely oxidized to TiO_2_.

Thermal-assisted strategy improves the aspect ratio of the structure and increases the oxidation of the surface, which further enhances the anti-reflectance performance of TC4.

### 3.4. Model and Application

**Figure 6:**
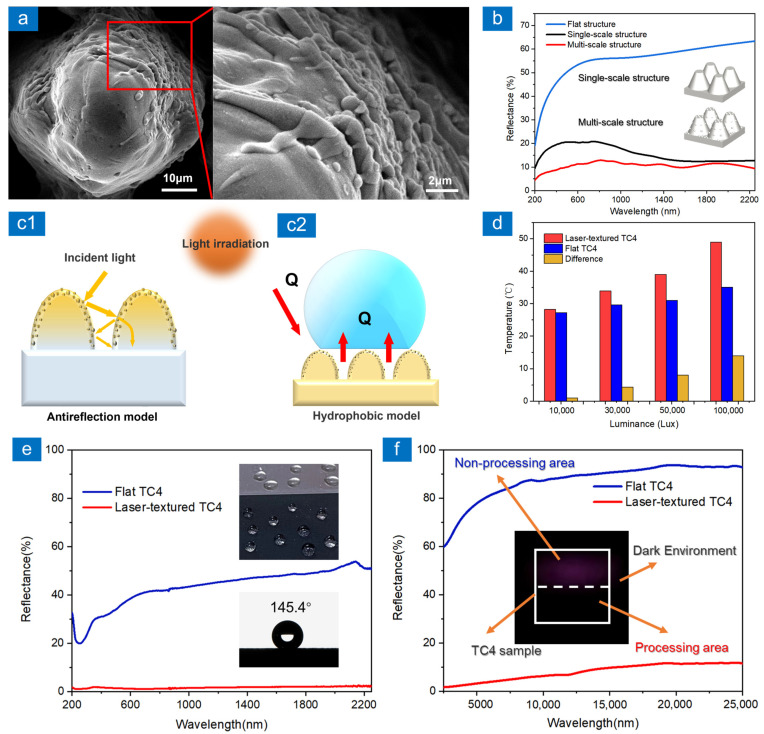
(**a**) Nanoparticles and nanotextures on multi-scale structured surfaces, (**b**) the reflectance simulations of single-scale and multi-scale structures by FDTD method, (**c**) the anti-reflection and wettability model of multi-scale structure, (d) the surface temperature increase of laser-textured TC4 and flat TC4 in the face of the same luminance, (**e**,**f**) the reflectance spectra of TC4 samples at different wavelengths, the static contact angle and the stealth effect of TC4 sample in dark environment. In addition, the laser-textured TC4 in Figure 6 is sample stru-120 (150 °C).

Figure 6a shows nanoparticles and nanotextures on multi-scale structured surfaces. Abundant nanostructures on the microcolumn surface are beneficial for anti-reflection. The reflectance simulations of single-scale and multi-scale structures by FDTD (Finite difference time domain) method are shown in Figure 6b. Due to the limitation of the computer memory, the bottom and upper diameter of the microcolumn in the single-scale structure is set to 10 μm and 5 μm, and the height is set to 15 μm. The multi-scale structure is formed by adding spheres with a diameter of 800 nm to the single-scale structure. In the simulation, the default level 2 mesh accuracy was adopted. It means that the generated mesh has at least 10 mesh points per wavelength. Figure 6b indicates the multi-scale structure has more excellent anti-reflection performance compared to the single-scale structure. When the incident light wavelength is less than or essentially equal to the size of the nanoparticle, the presence of nanoparticles can provide a larger light-trapping area. When the incident light wave is larger than the size of nanoparticles, the nanoparticles become sub-wavelength structures. According to the equivalent medium theory, it is known that the sub-wavelength structures provide a gradual change of refractive index to weaken the reflection caused by the sudden change of refractive index. As shown in Figure 6a, the size of micro-nano composite structure on the microcolumns surface ranges from 200 nm to 2000 nm. Depending on the relationship between nanoparticle size and incident light wavelength, nanoparticles can increase the light-trapping area or provide gradual refractive index, respectively. The anti-reflection model of the multi-scale structure is shown in Figure 6(c1). In addition to the nanoparticles, the adjacent microcolumns capture light and make it reflect many times in the structural gaps, thus enhancing light absorption. The higher light absorption results in a significant enhancement of the photothermal conversion on the material surface. The laser-textured TC4 in Figure 6 is sample stru-120 (150 °C). Figure 6d shows the surface temperature increase of laser-textured TC4 and flat TC4 in the face of the same luminance. The environmental temperature is 26 °C and the temperature rise of the laser-textured TC4 is significantly enhanced. Microcolumns with nanoparticles facilitate the capture of air pockets under the droplets and keep the droplets in the Cassie–Baxter wettability model, as shown in Figure 6(c2). The laser-textured TC4 without chemical treatment reached a static contact angle of 145.4° after 20 days in air, while the reflectance in the visible and near-infrared wavelengths was less than 2%, as shown in Figure 6e. The combination of high photothermal conversion and hydrophobicity is important for improving anti-icing performance of laser-textured TC4. In Figure 6f, the TC4 sample containing both laser processing and non-processing areas was placed in a dark environment with low light conditions. The non-processing area has obvious reflected light and is easily identified in the dark environment. In contrast, it is difficult to distinguish the laser processing area from the dark environment. Figure 6f additionally shows the reflection spectra of laser-textured TC4 at 2500–25,000 nm. Compared with the flat TC4, the reflectance of laser-textured TC4 is obviously reduced, which can achieve a stealth effect in the mid-infrared band.

## 4. Conclusions

In summary, a thermal-assisted strategy of laser processing ultra-low reflectance surface was proposed. This strategy combines the microcolumns produced by laser ablation with the recast layer produced by in situ deposition and Marangoni flow to fabricate multi-scale anti-reflection structure. The nanoparticles further reduce the surface reflectance. TC4 super-absorption surface (stru-120) with an average reflectance of only 2.10% at 200–2250 nm was prepared by adjusting the parameters of nanosecond laser. After that, the thermal-assisted strategy increases the aspect ratio of the microstructure and the surface oxygen content. This further improves the anti-reflection performance of the material. The stru-120 (150 °C) has an average reflectance of 1.71% at 200–2250 nm. The average reflectance of the stru-120 (150 °C) is 7.80% in the range of 2500–25,000 nm. To the best of our knowledge, this is the smallest reported value to date. However, the effect of external heat sources on the physical interaction between the laser and the material needs to be further investigated. In addition, laser-textured TC4 exhibits high efficiency for the absorption of solar energy and conversion to heat in the photothermal conversion experiment. The hydrophobic angle of the sample was 145.4° after standing for 20 days without chemical treatment. Low-cost nanosecond laser is convenient for large-area preparation of anti-reflection structures. All these advantages show this strategy to have a promising application in the preparation of broadband ultralow reflectance surface. Meanwhile, this strategy is prospective in the preparation of functional micro-nano composite structures, and can be applied to other surface engineering fields.

## Figures and Tables

**Table 1:** Atomic number percentage of Ti and O in different samples.

Sample	Ti (%)	O (%)
Stru-120 (26 °C)	38.23	61.77
Stru-120 (150 °C)	32.14	67.86

## Data Availability

The data presented in this study are available on request from the corresponding author.
